# New Perspective in Atrial Fibrillation

**DOI:** 10.3390/jcm9113713

**Published:** 2020-11-19

**Authors:** Audrey Sagnard, Nefissa Hammache, Jean-Marc Sellal, Charles Guenancia

**Affiliations:** 1 Cardiology Department, University Hospital, 21079 Dijon, France; audrey.sagnard@chu-dijon.fr; 2 Département de Cardiologie, Centre Hospitalier Universitaire (CHU de Nancy), 54500 Vandœuvre lès-Nancy, France; n.hammache@chru-nancy.fr (N.H.); jm.sellal@chru-nancy.fr (J.-M.S.); 3 INSERM-IADI U1254, 54500 Vandœuvre lès-Nancy, France; 4 PEC 2 EA 7460, University of Burgundy and Franche-Comté, 21000 Dijon, France

Despite a large number of publications on this subject, the pathophysiological mechanisms involved in atrial fibrillation (AF) onset and recurrence are uncertain. Moreover, though several thrombo-embolic and bleeding prediction [1] scores for AF patients have been developed, their performance is still limited [2]. Taken together, these facts suggest that we are still missing a global theory of atrial fibrillation pathophysiology (or at least some parts of it).

A better understanding of AF pathophysiology could come from the integration of all the cardiac environment modulators, including “Coumel triangle” components [3]. Indeed, apart from the pulmonary vein triggers that have been extensively studied [4], the relationship between other triggers (inflammation as in acute AF [5,6,7], stable coronary artery disease [8], or post-operative AF [9]), the modulator (mainly autonomic nervous system dysregulation [10]) and substrate alterations (fibrosis but also changes in the conduction properties of the atrial cells even in the absence of a quantifiable “scar” [11]) have been recently brought to light by several papers [12,13]. The interplay between cardiovascular risk factors, mainly high blood pressure [14] and obesity [6], atrial epicardial fat, and atrial ganglionated plexi [15], is complex and critical for the understanding of AF, but also in the search for new treatments. In this regard, a particular focus should be placed on the new anti-diabetic therapies (SGLT-2 inhibitors [16] and GLP-1 receptor agonists [17]) that have not only proven a benefit for major cardiovascular events (MACE) occurrence but also a decrease in AF burden. These treatments do not act so much as glycemia regulators, as Hb1Ac is usually only slightly decreased, but on the complex metabolic pathways involved in diabetic cardiomyopathy and possibly also in metabolic syndrome patients without diabetes. Thus, a fascinating field of research is open for the characterization of the metabolism’s role in AF onset and persistence and perhaps also as a therapeutic target, as suggested by the major results obtained through weight loss in obese patients suffering from AF [18].

In the past few years, the concept of atrial cardiopathy has emerged as a promising lead to connect AF to stroke, heart failure, and inflammatory processes; indeed, all of the mechanisms associated with atrial remodeling and the development of atrial cardiopathy are also likely to promote the development of AF [19]. An international expert consensus defined atrial cardiopathy as “any complex of structural, architectural, contractile, or electrophysiological changes affecting the atria with the potential to produce clinically-relevant manifestations” [20]. This recent concept suggests that the real trigger of stroke may be an abnormal atrial substrate rather than the atrial rhythm itself. Indeed, evidence from studies analyzing the data obtained from implantable cardiac devices has recently demonstrated that there is no temporal correlation between AF and stroke [21]. This finding is in line with current thinking on the pathophysiology of the relationship between AF and cardioembolic stroke [22]: AF seems to be more of a risk marker than the cardioembolic risk vector itself. It is, therefore, only a symptom of underlying atrial cardiomyopathy, which, even in sinus rhythm, increases thromboembolic risk. For now, however, the lack of a clinically validated definition of atrial cardiopathy limits its clinical applications and the reproducibility of the results obtained using these various definitions. Indeed, several clinical, electrocardiographic, biological, and imaging markers [23] have been suggested [24], but few of them have been correlated to atrial tissue abnormalities as defined by the international expert consensus [20].

The aim of this Special Issue is to gather basic research, as well as pathophysiological and epidemiological papers, focused on the relationship between atrial substrate and atrial fibrillation onset, recurrence, and outcomes.

## References

[1] Lopez-Minguez J.R., Nogales-Asensio J.M., De Oliveira E.I., Santos L., Ruiz-Salmeron R., Arzamendi-Aizpurua D., Costa M., Gutierrez-Garcia H., Fernandez-Diaz J.A., Freixa X. (2020). Major Bleeding Predictors in Patients with Left Atrial Appendage Closure: The Iberian Registry II. J. Clin. Med..

[2] Hirsh B.J., Copeland-Halperin R.S., Halperin J.L. (2015). Fibrotic atrial cardiomyopathy, atrial fibrillation, and thromboembolism: Mechanistic links and clinical inferences. J. Am. Coll. Cardiol..

[3] Coumel P. (1993). Cardiac arrhythmias and the autonomic nervous system. J. Cardiovasc. Electrophysiol..

[4] Haissaguerre M., Jais P., Shah D.C., Takahashi A., Hocini M., Quiniou G., Garrigue S., Le Mouroux A., Le Metayer P., Clementy J. (1998). Spontaneous initiation of atrial fibrillation by ectopic beats originating in the pulmonary veins. N. Engl. J. Med..

[5] Guenancia C., Binquet C., Laurent G., Vinault S., Bruyère R., Prin S., Pavon A., Charles P.E., Quenot J.P. (2015). Incidence and Predictors of New-Onset Atrial Fibrillation in Septic Shock Patients in a Medical ICU: Data from 7-Day Holter ECG Monitoring. PLoS ONE.

[6] Guenancia C., Stamboul K., Garnier F., Beer J.C., Touzery C., Lorgis L., Cottin Y., Zeller M. (2014). Obesity and new-onset atrial fibrillation in acute myocardial infarction: A gender specific risk factor. Int. J. Cardiol..

[7] Guenancia C., Toucas C., Fauchier L., Stamboul K., Garnier F., Mouhat B., Sagnard A., Lorgis L., Zeller M., Cottin Y. (2018). High rate of recurrence at long-term follow-up after new-onset atrial fibrillation during acute myocardial infarction. Europace.

[8] Ninni S., Lemesle G., Meurice T., Tricot O., Lamblin N., Bauters C. (2020). Real-Life Incident Atrial Fibrillation in Outpatients with Coronary Artery Disease. J. Clin. Med..

[9] Khan M.S., Yamashita K., Sharma V., Ranjan R., Dosdall D.J. (2020). RNAs and Gene Expression Predicting Postoperative Atrial Fibrillation in Cardiac Surgery Patients Undergoing Coronary Artery Bypass Grafting. J. Clin. Med..

[10] Sagnard A., Guenancia C., Mouhat B., Maza M., Fichot M., Moreau D., Garnier F., Lorgis L., Cottin Y., Zeller M. (2020). Involvement of Autonomic Nervous System in New-Onset Atrial Fibrillation during Acute Myocardial Infarction. J. Clin. Med..

[11] Guichard J.B., Nattel S. (2017). Atrial Cardiomyopathy: A Useful Notion in Cardiac Disease Management or a Passing Fad?. J. Am. Coll. Cardiol..

[12] Gaeta M., Bandera F., Tassinari F., Capasso L., Cargnelutti M., Pelissero G., Malavazos A.E., Ricci C. (2017). Is epicardial fat depot associated with atrial fibrillation? A systematic review and meta-analysis. Europace.

[13] Nalliah C.J., Bell J.R., Raaijmakers A.J.A., Waddell H.M., Wells S.P., Bernasochi G.B., Montgomery M.K., Binny S., Watts T., Joshi S.B. (2020). Epicardial Adipose Tissue Accumulation Confers Atrial Conduction Abnormality. J. Am. Coll. Cardiol..

[14] Park Y.J., Yang P.S., Yu H.T., Kim T.H., Jang E., Uhm J.S., Pak H.N., Lee M.H., Lip G.Y.H., Joung B. (2020). What Is the Ideal Blood Pressure Threshold for the Prevention of Atrial Fibrillation in Elderly General Population?. J. Clin. Med..

[15] Avazzadeh S., McBride S., O’Brien B., Coffey K., Elahi A., O’Halloran M., Soo A., Quinlan L.R. (2020). Ganglionated Plexi Ablation for the Treatment of Atrial Fibrillation. J. Clin. Med..

[16] Li W.J., Chen X.Q., Xu L.L., Li Y.Q., Luo B.H. (2020). SGLT2 inhibitors and atrial fibrillation in type 2 diabetes: A systematic review with meta-analysis of 16 randomized controlled trials. Cardiovasc. Diabetol..

[17] Nreu B., Dicembrini I., Tinti F., Sesti G., Mannucci E., Monami M. (2020). Major cardiovascular events, heart failure, and atrial fibrillation in patients treated with glucagon-like peptide-1 receptor agonists: An updated meta-analysis of randomized controlled trials. Nutr. Metab. Cardiovasc. Dis..

[18] Pathak R.K., Middeldorp M.E., Meredith M., Mehta A.B., Mahajan R., Wong C.X., Twomey D., Elliott A.D., Kalman J.M., Abhayaratna W.P. (2015). Long-Term Effect of Goal-Directed Weight Management in an Atrial Fibrillation Cohort: A Long-Term Follow-Up Study (LEGACY). J. Am. Coll. Cardiol..

[19] Guenancia C., Garnier F., Fichot M., Sagnard A., Laurent G., Lorgis L. (2020). Silent atrial fibrillation: Clinical management and perspectives. Future Cardiol..

[20] Goette A., Kalman J.M., Aguinaga L., Akar J., Cabrera J.A., Chen S.A., Chugh S.S., Corradi D., D’Avila A., Dobrev D. (2016). EHRA/HRS/APHRS/SOLAECE expert consensus on atrial cardiomyopathies: Definition, characterization, and clinical implication. Europace.

[21] Brambatti M., Connolly S.J., Gold M.R., Morillo C.A., Capucci A., Muto C., Lau C.P., Van Gelder I.C., Hohnloser S.H., Carlson M. (2014). Temporal relationship between subclinical atrial fibrillation and embolic events. Circulation.

[22] Calenda B.W., Fuster V., Halperin J.L., Granger C.B. (2016). Stroke risk assessment in atrial fibrillation: Risk factors and markers of atrial myopathy. Nat. Rev. Cardiol..

[23] Bernard A., Leclercq T., Comby P.-O., Duloquin G., Ricolfi F., Béjot Y., Guenancia C. (2020). High rate of cardiac thrombus diagnosed by adding cardiac imaging in acute stroke computed tomography protocol. Int. J. Stroke.

[24] Kamel H., Okin P.M., Longstreth W.T., Elkind M.S., Soliman E.Z. (2015). Atrial cardiopathy: A broadened concept of left atrial thromboembolism beyond atrial fibrillation. Future Cardiol..

